# Supplementary material for: WordCommentsAnalyzer: A windows software tool for qualitative research
Source: F1000Res. 2018 Sep 4;7:536. Originally published 2018 May 3. [Version 2] doi: 10.12688/f1000research.14819.2 (PMC6137411; doi:10.12688/f1000research.14819.2)
Supplement: Supplementary file 3 [file f1000research-7-17614-s0002.tgz › b2a79496-4ada-47c4-9682-a99ce867836d.zip!/Eight Iranian Health Journals/Journal of Knowledge and Health_42.docx]

© Journal of Knowledge and Health. This work is licensed under CC BY 4.0 (https://creativecommons.org/licenses/by/4.0/).

Volume & Issue: دوره 2، شماره 3: 1386

Title: ی سیستم نمره¬دهی آپاچی- دو در پیش¬بینی نتایج جداسازی از دستگاه تهویه مکانیکی

http://knh.shmu.ac.ir/index.php/site/article/view/241

مقدمه: عوارض نامطلوب متعددی که تهویه مکانیکی می­تواند به­همراه داشته باشد، پژوهش­گران را بر آن داشته است تا در جهت جداسازی به­موقع و سریع بیماران از دستگاه تهویه مکانیکی و پیش­بینی نتایج، سیستم­های نمره­دهی ICU را ارایه نمایند. در این میان سیستم نمره­دهی آپاچی- دو با استقبال عمومی بیش­تری همراه بوده است. در این پژوهش نقش سیستم نمره­دهی آپاچی- دو در پیش­بینی نتایج جداسازی از دستگاه تهویه مکانیکی در بیماران بستری ICU بیمارستان­های امام رضا (ع)، قائم (عج) و شهید کامیاب مشهد مورد بررسی قرار گرفت. مواد و روش­ها: این مطالعه توصیفی- مقطعی بر روی 300 بیمار بستری در ICU داخلی­- جراحی و تروما به روش نمونه­گیری مبتنی بر هدف و در یک دوره 6 ماهه صورت گرفت. جمع­آوری اطلاعات مربوط به انتخاب نمونه و مشخصات فردی با استفاده از پرسش­نامه و به روش ثبت از پرونده انجام شد. داده­های مربوط به فرم آپاچی- دو ناوس (1985) که شامل سه قسمت: نمره فیزیولوژیک حاد، تطبیق سن و تطبیق بیماری­های مزمن زمینه­ای می­باشد در دو نوبت: 24 ساعت اول پذیرش و زمان جداسازی کامل جمع­آوری گردید. نتايج: ارتباط معنی­دار آماری بین نمره آپاچی- دو در 24 ساعت اول پذیرش و زمان جداسازی کامل مشاهده گردید (به­ترتیب 005/0=P و 0001/0>P). هم­چنین برای سیستم نمره­دهی آپاچی- دو، حساسیت 81%، ویژگی80%، ارزش اخباری مثبت 79% و ارزش اخباری منفی 82% به­دست آمد. نتیجه­گیری: این مطالعه کارآیی مناسب سیستم نمره­دهی آپاچی- دو را در پیش­بینی نتایج جداسازی (نجات یافته، فوت شده) نشان می­دهد. امید است نتایج این پژوهش توجه مسئولین آموزشی، پژوهشی و مدیران را به امر جداسازی به­موقع و سریع­تر بیماران از دستگاه تهویه مکانیکی بر­اساس سیستم نمره­دهی مناسب­تر جلب نموده و در نتیجه شاهد ترخیص سریع و کم عارضه­تر و کاهش مرگ­و­میر بیماران در ICU باشیم.

Title: بررسي عوامل مؤثر بر بروز اشتباهات دارويي از ديدگاه پرستاران شاغل در بخش¬های مختلف بيمارستان امام حسين(ع) شاهرود

http://knh.shmu.ac.ir/index.php/site/article/view/242

مقدمه: اجراي دستورات دارویي بخش مهمي از فرآيند درمان و مراقبت از بيمار است و بروز اشتباهات دارویي يكي از تهديدات جدي در سلامت و ايمني بيمار و هم­چنين حرفه پرستاري محسوب مي­گردد. پژوهش حاضر با هدف تعيين عوامل مؤثر بر بروز اشتباهات دارويي از ديدگاه پرستاران صورت گرفته است.  مواد و روش­ها: در پژوهش توصيفي- تحليلي حاضر، تعداد 40 نفر از پرستاران شاغل در بخش­هاي مختلف بيمارستان امام حسين(ع) شاهرود به­صورت غيرتصادفي انتخاب و ديدگاه آنان در ارتباط با عوامل مؤثر در بروز اشتباهات دارويي با استفاده از پرسش­نامه طراحي شده، مورد سنجش قرار گرفت. برای بررسی اهمیت عوامل، هر گزینه با گزینه­های هیچ، کم، متوسط و زیاد، بر اساس میزان اهمیت از 0 تا 3 امتیازبندی گردید. سپس داده­ها وارد نرم­افزار SPSS گردید و داده­های کمی به­صورت میانگین و داده­های کیفی به­صورت درصد فراوانی نمایش داده شده و برای مقایسه گروه­ها از آزمون2 χ استفاده شده است. نتایج: با توجه به نتايج حاصله از اين پژوهش، اغلب واحد­هاي پژوهش، عواملي چون: خستگي ناشي از كار اضافي (7/66%)، كمبود تعداد پرستار نسبت به تعداد بيمار در بخش (59%)، مشکلات روحي و رواني پرستار (7/48%)، ناخوانا بودن دستور پزشك در پرونده بيمار (5/42%) و نداشتن وقت و فرصت كافي (1/42%) را از مهم­ترين عوامل مؤثر در بروز اشتباهات دارويي ذكر كردند. هم­چنين اغلب واحد­هاي پژوهش، عواملي چون: نحوه چيدن دارو در قفسه دارویي (4/47%)، شيوه نظارت و سرپرستي بخش (2/46%)، فضاي اتاق دارو (نور، فضاي فيزيكي و ...) (2/46%) و نحوه تجويز دارو (خوراكي، تزريقي و ...) (1/36%) را داراي كم­ترين اهميت در بروز اشتباهات دارويي برشمردند و عواملي چون: نوبت­كاري صبح (7/48%) و منشور دارویي (9/38%) را در بروز اشتباهات دارويي، بدون تأثير عنوان نمودند بين عوامل مؤثر بر بروز اشتباهات دارويي از دیدگاه پرستاران و متغيرهاي سن، جنس و سابقه کار رابطه معنی­داری مشاهده نشد. نتيجه‏گيري: با توجه به نتايج حاصل از پژوهش حاضر، مدیریت مؤثر، ارتقاء انگيزه در پرستاران و هم­چنين افزايش توان علمی و مهارت­های بالینی پرستاران در زمينه اجراي دستورات دارویي جهت پيش­گيري از بروز اشتباهات دارويي در بخش­هاي مختلف پيشنهاد مي‏گردد

Title: بررسي تريكومونيازيس در زنان مراجعه¬كننده به درمانگاه زنان و مراكز بهداشتي درماني شهر يزد در سال 85

http://knh.shmu.ac.ir/index.php/site/article/view/243

مقدمه: تریکوموناس واژینالیس تک­یاخته انگلی می­باشد که مسبب تریکومونیازیس در انسان می­باشد. این بیماری از بیماری­های منتقله از راه جنسی است که به­لحاظ انتشار جهانی، اهمیت دارد. این بیماری طیف وسیعی از نشانه­های بالینی از جمله التهاب شدید و سوزش توأم با ترشحات بد­بو و خارش را به­دنبال دارد. برآوردهای اخیر بیماری در جهان مؤید این است که بالغ بر 170میلیون نفر در جهان به این بیماری مبتلا هستند. هدف از انجام این تحقیق برآورد میزان شیوع و تعیین رابطه بین برخی علایم مؤثر نظیر عوامل دموگرافیک و نشانه­های بالینی بیماری بود. مواد و روش­ها: پژوهش حاضر مطالعه­ای مقطعی است که در آن 384 نفر از مراجعین به مراکز بهداشتی، درمانی شهر یزد در یک دوره یک­ساله توسط مامای آموزش دیده مورد معاینه و مصاحبه قرار گرفتند. در این معاینه علاوه بر مشاهده بالینی، دو نمونه با استفاده از سوآپ کتانی استریل از ناحیه فورنیکس خلفی و سرویکس برداشت شد. نتایج آزمایشات مربوط به گسترش مرطوب و کشت نمونه­ها و آزمون pH پس از درج در پرسش­نامه به­منظور تعیین شیوع و هم­بسته­های آن با استفاده از آزمون  χ2 تحلیل گردیدند. نتایج: در این مطالعه ملاحظه گردید که 8 نفر (1/2%) طی روش مشاهده مستقیم گسترش مرطوب و 10 نفر (6/2%) به روش کشت نمونه­ها مثبت تشخیص داده شدند. رابطه معنی­داری بین شغل افراد و ابتلا به بیماری دیده نشد (05/0P>)، ولی ارتباط معنی­داری بین شیوع ابتلا به تریکومونیازیس و تعداد دفعات زایمان به­دست آمد (003/0 P=). نتیجه­گیری: نتایج این پژوهش نشان داد که میزان شیوع تریکومونیازیس اگر چه نسبت به معدودی از پژوهش­ها افزایش اندکی یافته است اما نسبت به بسیاری از مطالعات دیگر در این زمینه کاهش چشمگیری را نشان می­دهد که به­نظر می­رسد عوامل دموگرافیک و فرهنگی بیش­ترین نقش را در بروز این کاهش ایفاد نموده­اند.

Title: درماتيت سيمان در كارگران صنايع ساختماني شاهرود و لردگان

http://knh.shmu.ac.ir/index.php/site/article/view/244

مقدمه: سيمان مدت­هاست که به­عنوان عامل ايجاد درماتيت تماسي تحريکي و آلرژيک شناخته شده است. از آنجا که مطالعات اندکي در مورد درماتيت شغلي در صنايع ساختمان ايران وجود دارد، هدف از اين مطالعه بررسي شيوع درماتيت سيمان و عوامل مؤثر بر آن در كارگران کارخانه سيمان و موزایيك­سازي­ها مي­باشد. مواد و روش­ها: نوع مطالعه توصیفی - مقطعی می­باشد. در این مطالعه برای 50 كارگر کارخانه سيمان شاهرود و 150 كارگر موزایيك­سازي در لردگان از طریق مصاحبه حضوري پرسش­نامه تکمیل گردید. پرسش­نامه شامل مشخصات دموگرافيک و سوالاتي در مورد ناراحتي پوستي در حال حاضر، سابقه اگزما، ساعات کار روزانه، مواد شيميايي مورد تماس، استفاده از دستکش و... بود. بيماران به­وسيله متخصص پوست مورد معاينه و آزمایش تماسی (Patch test) قرارگرفتند. تجزیه و تحلیل اطلاعات با استفاده از نرم افزار SPSS و آزمون­های آماری c2، آزمون t  و رگرسیون لجستیک انجام شد. نتایج: نتايج نشان مي­دهد 52 نفر (7/34%) در موزایيک­سازي­ها و 8 نفر (16%) در كارخانه سيمان درماتيت را در زمان مطالعه گزارش کردند. در موزایيک­سازي ها 5/15% به درماتيت آلرژيك مبتلا بودند. با افزايش سن، شيوع درماتيت دست در موزاييك سازي­ها افزايش يافته است. بين درماتيت و سابقه اگزما در كارگران موزاييك­سازي­ها ارتباط معني­داری مشاهده شد (05/0(P< . نتیجه­گیری: سيمان بايد به­عنوان يک ماده خطرناک در محيط کار تلقي شود. استفاده از دستکش مناسب، شناسايي افراد حساس در معاينات پيش از استخدام و آموزش بهداشت دوره­اي به كارگران توصيه مي­گردد.

Title: بررسي ميزان دانش، نگرش و مهارت پرستاران شاغل در بخش¬هاي مراقبت¬هاي ويژه جراحی قلب باز در پيش¬گيري از نشان¬گان ICU

http://knh.shmu.ac.ir/index.php/site/article/view/245

مقدمه: نشان­گان ICU حالت­هاي هيجاني و عاطفي است كه در محيط­هاي تنش­زاي بخش­هاي ويژه به اشكال دليريوم، سايكوز و يا نوروز رخ مي­دهد. علایم ويژه دليريوم كه در برخي منابع به نام سايكوز ICU ناميده مي­شود، شامل بي­خوابي، بي­قراري، عدم آگاهي، تخريب حافظه، توهمات شنوايي، هذيان گزند و آسيب و ترس و واهمه است. اين پژوهش يك مطالعه توصيفي- تحليلي مي­باشد كه با هدف تعيين ميزان دانش، نگرش و مهارت پرستاران شاغل در بخش­هاي مراقبت­هاي ويژه(ICU) در پيش­گيري از بروز نشان­گان ICU در بيمارستان­هاي وابسته به وزارت بهداشت درمان و آموزش پزشكي در شهر تهران انجام گرفته است. مواد و روش­ها: جامعه پژوهش شامل كليه پرستاراني بود كه در زمان اجراي پژوهش در يكي از بخش­هاي ICU جراحي قلب باز در بيمارستان­هاي وابسته به وزارت بهداشت، درمان و آموزش پزشكي در شهر تهران شاغل بودند. به­دليل گستردگي ICU جراحي قلب باز در تهران نمونه­گيري به روش خوشه­اي انجام شد و كليه پرستاران (56 نفر) شاغل در خوشه­هاي مورد نظر به­عنوان نمونه­هاي پژوهش در نظر گرفته شدند. نتایج: با استفاده از آزمونc2، ارتباط ميان دانش، نگرش و مهارت­هاي واحد­هاي پژوهش با هر يك از متغير­هاي سن، جنس، ميزان تحصيلات، وضعيت تأهل، سابقه كار، سابقه كار در ICU و گذرانیدن دوره مراقبت­هاي ويژه رابطه معني­داري با افزايش سطح دانش نمونه­های پژوهش داشته است. در­حالي­كه هيچ­يك از متغيرهاي سن، وضعيت تأهل، جنس، ميزان تحصيلات، سابقه كار و سابقه كار در بخش­هاي ويژه، رابطه معني­داري با ميزان نمونه­ها نداشتند. همچنين يافته­هاي اين پژوهش وجود هر­گونه ارتباط ميان نگرش نمونه­ها و متغير­هاي سن، جنس، وضعيت تأهل،‌ ميزان تحصيلات، سابقه كار و سابقه كار در بخش­هاي ويژه را رد مي­كند. نتیجه­گیری: نتايج تحقيق مؤيد آن است كه ميان ميزان تحصيلات و گذرانیدن دوره مراقبت­هاي ويژه با ميزان مهارت پرستاران در پيش­گيري از نشان­گان ICU رابطه وجود دارد. پرستاران شاغل در ICU نيازمند آموزش مداوم در زمينه راه­هاي پيش­گيري و مقابله با نشان­گان ICU مي­باشند. هم­چنين ساختار فيزيكي بسياري از بخش­هاي ويژه امروزي در كشور ما نيازمند تغیيراتي مي­باشد تا  به  اين وسيله امكان برقراري ارتباط بيش­تري بين بيمار و اعضای خانواده وي در مدت بستري بودن در بيمارستان و ICU فراهم گردد.

Title: عوامل خطر پوکی استخوان در زنان یائسه شهری

http://knh.shmu.ac.ir/index.php/site/article/view/246

قدمه: امروزه پوکی استخوان و شکستگی­های ناشی از آن یک مشکل بزرگ بهداشتی در سنین کهن­سالی می­باشد. این عارضه در خانم­ها و در سنین پس از یائسگی بسیار شایع است. با توجه به کمبود اطلاعات و تحقیقات در خصوص پوکی استخوان در کشورهای در حال توسعه، تحقیق فوق به­منظور بررسی عوامل خطر پوکی استخوان در خانم­های یائسه در شهر تهران صورت گرفته است. مواد و روش­ها: این تحقیق یک مطالعه مورد- شاهدی است که در مرکز سنجش تراکم استخوان بیمارستان شریعتی طی سال­های 83-80 انجام گرفته است. در این تحقیق 272خانم یائسه (136 خانم دچار پوکی استخوان و 136 خانم را به­عنوان شاهد با همان نسبت از نظر گروه سنی) مراجعه­کننده به مراکز سنجش استخوان مورد مصاحبه قرار گرفتند. تشخیص پوکی استخوان با استفاده از روش DEXA و دستگاه سنجش تراکم استخوان Lunar DPX و بر اساس تعریف WHO در ناحیه ستون مهره­ها و یا استخوان سر ران انجام گرفت. جمع­آوری اطلاعات از طریق تکمیل پرسش­نامه به­وسیله مصاحبه و ثبت نتایج آزمایشات انجام شد. پرسش­نامه حاوی اطلاعات دموگرافیک سابقه بیماری، استفاده از داروها و مکمل­های غذایی، وضعیت تغذیه، اطلاعات مربوط به بارداری­های قبلی و فعالیت فیزیکی بود. عوامل خطر با استفاده از رگرسیون لجستیک چندگانه و برآورد نسبت شانس مورد بررسی قرار گرفت. نتایج: عوامل خطر پوکی استخوان در جمعیت مورد مطالعه با نسبت­های شانس تعیین شده (05/0P<) عبارت بودند از طول دوره یائسگی بیش از 5 سال (4/2)، BMI کم­تر از 25 (2/4)، استفاده از استروییدها (6/3)، اختلالات مفصلی و استخوانی (2/2)، مشکلات دندان­ها (3/2)، تعداد زایمان بیش از 3 (1/2)، شیردهی بیش از 2 سال (6/2)، تحصیلات کم­تر از دیپلم (4/2) و مصرف گوشت قرمز بیش از 4 وعده در هفته (1/2) .در حالی­­که مصرف منظم (روزانه30 گرم یا بیش­تر) پنیر (5/0)، مصرف منظم (سه وعده یا بیش­تر در هفته) شیر )4/0(، مرغ (4/0)، ماهی (3/0)، عسل (42/0) با کاهش خطر پوکی استخوان همراه بود. هم­چنین مصرف منظم قرص­های مکمل کلسیم (3/0)، پیاده­روی (4/0) و قرار گرفتن در معرض نور مستقیم آفتاب (4/0) به­عنوان عوامل پیش­گیری کننده از پوکی استخوان مطرح گردیدند. نتیجه­گیری: بر اساس نتایج به­دست آمده از تحقیق حاضر، پوکی استخوان با عوامل متعدد و شناخته شده خطرساز همراه بود. .با توجه به تأثیر عوامل محیطی مثل تغذیه، ورزش و قرار گرفتن در معرض نور آفتاب بر پوکی استخوان، انجام تحقیقات تکمیلی در این خصوص و اجرای برنامه­های آموزشی در زمینه تغذیه صحیح، ورزش و تغییر سبک زندگی به­منظور پیش­گیری از این عارضه ضروری به­نظر می­رسد.

Title: بررسي شيوع و علل حوادث منجر به بستري در مراجعين به بخش اورژانس بيمارستان امام حسين(ع) شاهرود

http://knh.shmu.ac.ir/index.php/site/article/view/247

مقدمه: حوادث به­عنوان دومين علت معلوليت­ها ­و يكي از مشكلاتي است كه مي­تواند سبب اختلالات جسمي و روحي- رواني و در موارد شديدتر منجر به مرگ گردد. اين پژوهش به­منظور تعيين ميزان شيوع و علل حوادث منجر به بستري در مراجعين به بخش اورژانس بيمارستان امام حسين(ع) شاهرود وابسته به دانشگاه علوم پزشكي انجام شده است. مواد و روش­ها: پژوهش حاضر يك مطالعه مقطعي- تحليلي است كه بر روي 3027 نفر از مصدومين ناشي از حوادث مختلف مراجعه كننده به بخش اورژانس بيمارستان امام حسين (ع) شاهرود صورت گرفته است. اطلاعات لازم توسط پرسش­نامه و از طريق مطالعه پرونده‏هاي بايگاني شده در طول يك­سال در بيمارستان جمع‏آوري گرديد و داده­ها با استفاده از آمار توصيفي، آزمون t مستقل، آناليز واريانس يك­طرفه و ضريب همبستگي پيرسون مورد تجزيه و تحليل و مقايسه قرار گرفت. آزمون‏هاي فوق توسط نرم­افزار SPSS تحت ويندوز انجام گرديد. نتايج: بر اساس نتايج حاصل از اين پژوهش، از كل 3027 حادثه منجر به بستري، 7/73% مربوط به مردان و 3/26% مربوط به زنان بوده است. هم­چنين بيش­ترين موارد حادثه در سن 15 تا 20 سال بود و بيش­ترين حادثه در افراد متأهل رخ داده بود. طبق نتايج، بيش­تر حوادث (42%) را سوانح ترافيكي تشكيل مي‏دهند و از 3/51% حوادث كه محل آنها مشخص شده، 4/83% آن در خيابان رخ داده كه بيش­ترين آن­ها در فصل تابستان و در ماه شهريور و در هنگام عصر اتفاق افتاده است. در اين پژوهش بين نوع حادثه و سن، جنس، محل حادثه، فصل حادثه، ماه و زمان (ساعت) حادثه ارتباط معني‏دار وجود داشت (05/0 P<)، ولي بين نوع حادثه و روز حادثه ارتباط معني‏دار مشاهده نگرديد (05/0 P>). نتيجه‏گيري: حوادث همانند بسياري از بيماري­ها قابل پيش­گيري است و اين خود مستلزم توسعه آگاهي مردم نسبت به رعايت اصول ايمني در هنگام رانندگي و افزايش ضريب ايمني خيابان­ها و جاده‏ها مي‏باشد. برنامه‏ريزي مؤثر درماني و مراقبتي در مراكز و بخش­هاي اورژانس در كاهش مرگ و­مير ناشي از حوادث بسيار اساسي است.

Title: مقایسه نتایج سنجش سرمی LDL-C به روش آنزیماتیک با روش محاسباتی فرید والد، دلونگ و فرید والد تعدیل شده

http://knh.shmu.ac.ir/index.php/site/article/view/248

مقدمه: کاهش کلسترول به­ويژه LDL-C از اهداف اصلي در بيماران مبتلا به بيماري­هاي عروق کرونر مي­باشد. اولين قدم، تغيير رفتار و سبك زندگي فرد است مانند تغيير رژيم غذايي، كاهش وزن، ورزش و در قدم دوم استفاده از داروهاي پايين آورنده ليپيدهاي خون است. اندازه­گيري مستقيم LDL روشي مناسب ولي گران مي­باشد. در اكثر آزمايشگاه­ها از روش محاسباتي فريدوالد استفاده مي­شود که تحت تاثيرعوامل مختلفي از قبيل بالا بودن سطح تري­گليسيريد، ضرورت ناشتا بودن و تأثيرپذيري از ضريب تغييرات آناليز ديگر متغيرها، قرار مي­گيرد. مطالعه حاضر با هدف مقايسه روش آنزيماتيك Wako با روش­هاي محاسباتي فريدوالد، فريدوالد تعديل شده و دلونگ براي سنجش سطح سرمي LDL-C طراحي شده است. مواد و روش­ها: در اين مطالعه 475 نمونه سرمي ناشتا مشتمل بر نمونه سرم 199 نفر مرد و 276 نفر زن با ميانگين سني 3/56 سال جهت تعيين LDL-C به روش آنزيماتيک بررسي شده است. جمعيت مورد مطالعه را بيماران بيمارستان امام حسين(ع) شاهرود تشکيل داده که مقدار تري گليسيريد آنها کم­تر از mg/dl 400 مي­باشد. بر حسب مقاديرTG نمونه ها به 4 همگروه (100>، 199-100، 299-200 و 400-300) تقسيم شده است. با استفاده از روش­هاي محاسباتي فريدوالد، فريدوالد تعديل شده و دلونگ مقادير سرمي LDL محاسبه و با نتايج روش آنزيماتيک با استفاده از نرم افزار Table Curve و SPSS تجزيه و تحليل شده است. نتايج: بررسي تفاوت بين مقادير LDL به­روش آنزيماتيک با روش­هاي محاسباتي نشان مي­دهد: 1- ميزان هم­بستگي در فاصله 20±0 ميلي­گرم در دسي­ليتر، پايين مي­باشد. 2- روش فريدوالد از هم­بستگي بالاتري نسبت به دلونگ و فريدوالد تعديل شده برخوردار مي­باشد (2/51% در مقابل 7/38% و 7/42%). 3- روش­هاي محاسباتي باعث ايجاد بيش­تخميني مقدار LDL مي­شوند (ميانگين روش فريدوالد mg/dl 7/17 بيش­تر از روش آنزيماتيک). 4- با افزايش مقادير تري­گليسيريد، ميزان هم­بستگي کاهش و بيش­تخميني LDL افزايش مي­يابد. ضريب هم­بستگي پيرسون 66/0 مي باشد (05/0 p<). نتيجه­گيري: با توجه به اهميت اندازه­گيري LDL در بيماري­هاي عروقي و نتايج مشاهده شده که در بيش از 50 درصد نمونه­ها، قدر مطلق تفاوت مقادير LDL بيش از mg/dl 20 مي­باشد، پيشنهاد مي­شود در اين بيماران براي انجام مداخله از روش آنزيماتيک استفاده شود.
